# Advancing Digital Medicine with Wearables in the Wild

**DOI:** 10.3390/s22124576

**Published:** 2022-06-17

**Authors:** Ryan S. McGinnis, Ellen W. McGinnis

**Affiliations:** 1 M-Sense Research Group, College of Engineering and Mathematical Sciences, University of Vermont, Burlington, VT 05405, USA; 2 Department of Psychiatry, Larner College of Medicine, University of Vermont, Burlington, VT 05405, USA; ellen.mcginnis@uvm.edu

This editorial provides a concise overview of the use and importance of wearables in the emerging field of digital medicine. We discuss best practices for evaluation of these technologies and briefly highlight several exciting areas where wearables are enabling novel insights and have the potential to transform medical care. Finally, for the reader’s guidance, we give a succinct overview of the papers included in this Special Issue and place them in the context of the best practice evaluation framework.

The emerging field of digital medicine aims to leverage advances in wearable and mobile technology to have a direct impact on diagnosing, preventing, monitoring, and treating disease [1]. The digital medicine revolution is at the cusp of transforming biomedical research and clinical practice by providing unprecedented access to ecologically valid health data and the ability to deliver personalized, data-informed, health-improving interventions directly to the patient wherever and whenever needed.

In the context of digital medicine, we often think of three primary areas of technical development: digital biomarkers, digital phenotypes, and digital therapeutics. Digital biomarkers use data from cutting-edge wearable sensors and mobile phones [2,3] to capture objective information about a patient’s physiological and/or behavioral status and potentially over long periods of time outside of research or clinical contexts. These digital biomarkers can be combined, sometimes using advanced computational approaches (e.g., via artificial intelligence or machine learning), to discover digital phenotypes of disease that can improve clinical assessment. Finally, digital therapeutics leverage wearable and mobile devices to deliver interventions to patients, with cutting-edge examples informing or personalizing those interventions based on a patient’s digital phenotype. In developing digital biomarkers, phenotypes, and therapeutics, digital medicine research discovers fundamental physiological processes underlying disease while advancing innovative technologies that will transform clinical practice and improve human health. Importantly, these advances enable the delivery of healthcare at scale, and potentially in ways that address healthcare disparities arising from accessibility challenges due to geographic location or socioeconomic status (e.g., [4]).

However, before this vision can be realized, it is critical that digital medicine technologies undergo rigorous evaluation [1]. Despite this need, validation efforts are fractured, with recent calls for a structured process for validation that encompasses technical, clinical, and healthcare-system-level considerations [5,6].

## 1. Evaluation Framework for Digital Medicine Technologies

A compelling evaluation framework is emerging, through the efforts of the Digital Medicine Society [7] and others, that aims to identify digital medicine technologies that are fit for purpose (e.g., see [5,6]). The comprehensive framework incorporates Measurement Verification, Analytical Validation, Clinical Validation, and Clinical Utility testing to help foster the development of digital medicine technologies that function as designed and markedly improve human health. The framework is structured to provide evaluation at every stage of the development process, starting with verification that sample-level sensor outputs meet pre-defined design criteria (Measurement Verification). From there, Analytical Validation establishes the performance of the algorithms acting to translate the raw sensor data into measures of human physiology or behavior. The next step is Clinical Validation, which demonstrates that a digital medicine technology captures the phenotype of interest in the intended clinical population. Finally, Clinical Utility testing evaluates if the technology addresses the needs of end users including safety of the technology and if it leads to improved health outcomes (efficacy) or provides useful information about the diagnosis, treatment, or prevention of a disease. Importantly, for a digital medicine tool to be clinically useful, it must also deliver value for a variety of stakeholders across the healthcare ecosystem so that engagement is maintained and health improvements can be realized. In the case of wearables, clinical utility includes the convenience and wearability of devices, as well as the helpfulness and usability of associated apps. Both devices and apps should be well-designed to deliver value to patients, providers, and other stakeholders throughout the healthcare system.

## 2. Overview of Special Issue on Applications of Wearables in Digital Medicine

The articles in this Special Issue have made significant contributions to the field of digital medicine with findings predominantly in the evaluation areas of Measurement Verification, Analytical Validation, and Clinical Validation. The papers present new wearable technologies and algorithms, evaluate new uses of existing consumer technologies, and begin to examine potential confounding factors when considering wearables data collected in the wild.

Articles that make contributions in the area of Measurement Verification and Analytical Validation introduce new wearable sensor technologies and evaluate their suitability for use in digital medicine. For example, one paper introduces SensorHub [8], which provides a framework for combining data from consumer-grade wearables to enable multimodal observational studies. Other papers present detailed evaluations of connected insoles for quantifying mobility impairment outside of the clinic [9] and a wrist wearable for robust activity recognition and energy expenditure estimation in older adults with high and low physical function [10]. Three others present the evaluation of technologies that enable ambulatory electrocardiogram (ECG) monitoring [11,12,13] for capturing atrial fibrillation and general arrhythmias, and even with three-lead ECG [13], providing the data necessary to inform treatment for these conditions. New machine-learning-based methods are introduced for hydration monitoring [14] from multiple wearable sensing modalities and estimating continuous blood pressure from photoplethysmography data [15]. Two additional papers also address challenges inherent to deploying wearables in the wild. One leverages temperature measurements to provide accurate differentiation between non-wear time, sleep time, and sedentary wake time [16]. The other demonstrates the importance of context in interpreting gait variability measures in persons with multiple sclerosis [17].

There is one article in this Special Issue that contributes a Clinical Validation. This article introduces a new algorithm for characterizing sit–stand transfer performance in the wild that needs only data from a single inertial sensor secured to the lower back [18]. Importantly, this method is extensively validated in younger and older healthy adults as well as in people with Parkinson’s disease. The authors also show that measurements taken in the wild differ from those captured in the lab, three days of data are required to provide reliable estimates of the performance metrics, and that estimates from the wild show larger group differences than those taken in lab. This study addresses critical issues impacting the deployment of wearables for digital medicine and the analysis of the resulting data for characterizing balance and mobility impairment.

## 3. Areas of Opportunity for Digital Medicine

Measurement Verification, Analytical Validation, Clinical Validation, and Clinical Utility testing help drive progress in Digital Medicine, enabling better access to objective assessment, via the passive measurement of patient behavior and physiology, and personalized care than can be delivered asynchronously and potentially automatically when it is most needed. We have identified several clinical areas where this approach may have the most benefit and thus could serve as compelling initial use cases for these approaches, including: for conditions where symptoms cannot be reported reliably, for episodic conditions, and for conditions with rapidly changing and/or context-dependent symptoms.

Digital medicine technologies and wearables can make a significant impact on conditions wherein symptoms cannot be reported reliably. For example, children under 8 years old are not able to accurately describe abstract emotions and thus are unable to report their own mental health problems [19,20,21,22,23], and adults with dementia [24] or psychosis have an impaired capacity to reliably report on their symptoms [25,26] as a product of the disease. In these cases, digital medicine technologies can capture objective metrics of their symptoms and convey that information directly to clinical decision makers. Digital phenotypes can give voice to those whose symptoms may be unintentionally overlooked by caregivers and providers, especially when symptoms are unobservable (i.e., feelings of worthlessness, confusion, and/or voice-hearing). As objective diagnostic markers are just now emerging in the fields of mental health and neurocognition, digital medicine is becoming more accessible for informing diagnostic and treatment decisions.

Another critical area where digital medicine can make a significant impact is for episodic health problems by enabling the objective tracking of episodes and predicting risk. While individuals with episodic conditions may be able to report their symptoms and even seek help during the episode, an accurate characterization of these symptoms is often difficult, and treatment is often necessary immediately or better prescribed as a preventative measure. For instance, conditions such as experiencing panic attacks [27,28], mania [29,30], suicidality [31,32], epileptic seizures [33], or atrial fibrillation [11,34] would all benefit from objective characterization of frequency, severity, and duration to inform appropriate care. Moreover, while these conditions may feel unpredictable, emerging data suggests digital markers may indicate elevated risk for an episode. Having even a few minutes or hours of warning would allow patients and clinical decision makers precious time to plan for, prevent, and/or intervene on coming episodes.

A third critical area where digital medicine can make a significant impact is for health conditions with rapidly changing and/or context-dependent symptoms, where symptoms cannot be captured well in a single clinic visit. In these cases, treatment would benefit from remotely and continuously characterizing these changes [35,36,37,38,39]. For example, markers of balance and mobility impairment often differ between clinic and free living environments and can change rapidly [18,40,41,42]. Similarly, eating disorders [43] and substance use disorders [44] are often associated with context-dependent triggers that continuously change in severity and immediate treatment needs. For these populations, digital therapeutics [45] that help assess and intervene when risk is high may be especially beneficial.

In summary, digital medicine technologies can offer objective assessment based on digital biomarkers and phenotypes to better identify vulnerable populations and digital therapeutics that detect when risk is high and offer the appropriate treatment. Deploying wearables “in the wild” enables these benefits of digital medicine, empowering patients by providing tools to those who need them, when they need them the most. This Special Issue provides several excellent examples of new wearable sensor technologies and associated algorithms well on their way to being fit for use in digital medicine. Future opportunities exist for further evaluation of these technologies and in clinical areas that would benefit from objective assessment of symptoms that cannot otherwise be reported reliably, objective tracking of episodes and predicting risk, and remote patient monitoring for the delivery of personalized therapeutics when and where they are most needed.
